# Analysis of Cough Factors and Quality of Life Score Among Children With Protracted Bacterial Bronchitis: Cross-Sectional Study

**DOI:** 10.2196/82887

**Published:** 2025-12-19

**Authors:** Haonan Ning, Wenyu Zheng, Jinghui Zhang, Fuhai Li, Nana Qiao

**Affiliations:** 1 Department of Pediatrics, Qingdao Central Hospital, University of Health and Rehabilitation Sciences, Qingdao, China; 2 Department of Pediatrics, Linyi People’s Hospital, Linyi, China; 3 Department of Pediatrics, Qilu Hospital of Shandong University, No. 107, Wenhua West Road, Lixia District, Jinan, Shandong Province, 250012, China, 86 18560086322

**Keywords:** protracted bacterial bronchitis, duration of cough, sCSS, PC-QOL, LCQ-MC, simplified Cough Symptom Score, Parent-Proxy Cough-Specific Quality of Life, Leicester Cough Questionnaire in Mandarin-Chinese

## Abstract

**Background:**

Protracted bacterial bronchitis (PBB) is a leading cause of chronic wet cough in children. Misdiagnosis and inadequate treatment may lead to the progression of diseases.

**Objective:**

The objective of this paper was to analyze factors influencing the cough duration prior to the diagnosis and assess health-related quality of life in children with PBB.

**Methods:**

Children diagnosed with PBB in the Qilu Hospital of Shandong University from November 2021 to November 2022 were included in this study. Clinical data were collected; parents completed the Parent-Proxy Cough-Specific Quality of Life (PC-QOL) questionnaire and the simplified Cough Symptom Score. Children aged 6 years and older completed the Leicester Cough Questionnaire in Mandarin-Chinese (LCQ-MC).

**Results:**

As of November 2022, we enrolled 88 patients. Place of residence (B=9.35, 95% CI 0.36-18.35; *P*=.04) and rest status during the coughing episode (B=7.87, 95% CI 0.36-15.38; *P*=.04) were significantly associated with cough duration prior to the diagnosis. PC-QOL scores (physical: mean 3.10, SD 1.36; psychological: mean 3.32, SD 1.57; social: mean 3.67, SD 1.53; total: mean 10.09, SD 4.21) showed physical-social differences (*t*_174_=−2.58, *P*=.01). PC-QOL posttreatment scores were significantly higher than pretreatment scores (physical: *t*_18_=–6.05, *P*<.001; psychological: *t*_18_=–4.42, *P*<.001; social: *t*_18_=−4.79, *P*<.001; total: *t*_18_=–5.25, *P*<.001). However, the scores of each PC-QOL domain were significantly lower than those of the LCQ-MC (physical: *t*_34_=8.31, *P*<.001; psychological: *t*_34_=6.58, *P*<.001; social: *t*_34_=5.09, *P*<.001; total: *t*_34_=8.11, *P*<.001).

**Conclusions:**

Place of residence and rest status during the coughing episode were significantly associated with cough duration prior to the diagnosis**.** Furthermore, PBB significantly reduces quality of life in physical, psychological, and social aspects.

## Introduction

Cough is a prevalent symptom of respiratory disorders among children, with children presenting with cough constituting 70% of primary complaints in outpatient visits to pediatric respiratory clinics. Notably, chronic cough, defined as a cough lasting more than 4 weeks, accounts for 10% of these outpatient cases [1-3]. Chronic cough in children originates from various factors, and its etiology varies based on the child’s age. Common causes of cough include respiratory infections, postinfectious cough, cough variant asthma, upper airway cough syndrome, and protracted bacterial bronchitis (PBB) [4-6].

PBB was initially proposed in 2006 [7], representing a chronic bacterial infection of the endobronchial membrane. This condition manifests as a persistent wet cough in children and may account for up to 42% of chronic cough cases [8,9]. Studies have indicated that the protracted duration of chronic cough is linked to respiratory dysfunction, impaired lung function, and potential development of bronchiectasis [10-13]. The incidence of cough-related complications escalates with both the prolongation of the disease and increased cough frequency, with a more than 2-fold increase in complication rates when symptoms persist beyond 8 weeks [14]. Inadequate treatment of PBB can result in irreparable damage to the bronchial mucosa, potentially progressing into chronic suppurative lung disease or bronchiectasis [15]. A recent study [16] indicates that a significant proportion of children experience respiratory symptoms many years later; some have an objectively reduced lung function and structural changes of the bronchial wall despite adequate initial therapy. Additionally, PBB profoundly affects the quality of life of both affected children and their families [17].

Therefore, PBB not only affects the physical health of children but also undermines their psychological, social, and familial functioning. The Clinical Practice Guide for Diagnosis and Treatment of Cough in Chinese Children underscores the significance of pediatricians in China focusing on children’s quality of life, advocating for the development and validation of a Cough-Specific Quality of Life Scale tailored for domestic children [18]. According to the 2015 Chest Guidelines [19], the Leicester Cough Questionnaire (LCQ) [20] and Parent-Proxy Cough-Specific Quality of Life (PC-QOL) questionnaire [21] have emerged as reliable and efficacious metrics for assessing quality of life in children experiencing chronic cough. Presently, there are few reports addressing the quality of life of children experiencing PBB. Highlighting this aspect of cough management is crucial for raising clinical awareness. To gain a deeper insight into PBB, this study dissected the clinical profiles of children with PBB, delved into the reasons behind their protracted disease trajectory, and evaluated the detrimental effects of chronic cough on their quality of life.

## Methods

### Participants

A comprehensive study was conducted on 88 pediatric patients diagnosed with PBB, who were admitted to the Pediatrics Department of Qilu Hospital, Shandong University, between November 2021 and November 2022. In total, 88 pediatric patients were included from outpatient and inpatient departments, and we obtained written consent from their guardians. All enrolled participants must meet the diagnostic criteria for PBB established by the American College of Chest Physicians (CHEST) Guidelines and Expert Panel [22].

The relevant definitions are as follows:

- Clinically based PBB: children aged ≤14 years with chronic wet or productive cough unrelated to an underlying disease and without any specific cough pointers (eg, coughing with feeding and digital clubbing) and whose cough resolves within 2 weeks of treatment with antibiotics targeted to local antibiotic sensitivities.
- Microbiologically based PBB: children aged ≤14 years with PBB with lower airway (bronchoalveolar lavage or sputum) confirmation of clinically important density of respiratory bacteria (≥10^4^ CFU/mL).

The exclusion criteria were as follows: (1) the presence of acute respiratory symptoms, such as fever or rapid breathing before enrollment; (2) chest X-ray or chest CT scans indicating pneumonia after hospitalization; (3) specific cough symptoms or signs, such as chest pain, suspected history of foreign body inhalation, hemoptysis, repeated sinus lung infections, history of tuberculosis exposure, dyspnea, chest deformities, wet rales on lung auscultation, changes in chest imaging, abnormal lung function, etc; (4) the coexistence of severe underlying diseases, including but not limited to neuromuscular disorders, immunodeficiency, chronic corticosteroid use, congenital heart disease, and delayed growth and development; and (5) inability to obtain written informed consent from the children or their legal guardians.

### General Information

The characteristics of the included children, such as their age, sex, duration of coughing prior to the diagnosis, characteristics of their cough (eg, wet, dry, or accompanied by wheezing), residential area, the presence of siblings, their parents’ age and educational attainment, their primary caregiver’s status, underlying health issues, exposure to smoking environments, and previous medical diagnoses and treatments, were comprehensively collected. Furthermore, the diagnoses and treatments provided to the children both in and out of the hospital during our research, including the type of antibiotics, length of treatment, and the efficacy of antibiotic therapy (Multimedia Appendix 1). For pediatric patients with PBB who showed inadequate response to empirical antibiotic therapy administered externally, electronic bronchoscopy was performed after hospitalization to identify pathogens and rule out potential underlying diseases. The pathogens found in bronchoalveolar lavage fluid were recorded.

### Simplified Cough Symptom Score

The simplified Cough Symptom Score (sCSS; Table S1 in Multimedia Appendix 2) [23], which is determined based on the description of the frequency and intensity of daytime and nocturnal coughing, and its effect on daily activities and sleep patterns, is categorized into 4 distinct grades based on symptom severity. This scale is a simplified version developed based on the Cough Symptom Score (Table S2 in Multimedia Appendix 2). Research [24] shows that the sCSS is a reliable and valid instrument for assessing cough symptoms. A higher sCSS indicates a more severe cough, which negatively affects children. Using the sCSS obtained during the initial visit, guardians were subsequently contacted through outpatient follow-ups and telephone interviews to monitor the fluctuations in the sCSS over 2 and 4 weeks of treatment, including at night. The scores were interpreted as follows:

1. A score of 0 meant the complete resolution of cough symptoms.
2. A decrease in the sCSS compared to the initial visit without reaching 0 indicated improvement in the cough symptoms.
3. No change in sCSS meant no significant change in cough symptoms.
4. An increase in the sCSS compared to the initial visit indicated a worsening of the cough symptoms.

### The Quality of Life Scale

The Leicester Cough Questionnaire in Mandarin-Chinese (LCQ-MC) [25] and PC-QOL questionnaire [21] were used to evaluate the impact of cough and disease experience on both children’s and parents’ overall quality of life (Tables S3-S5 in Multimedia Appendix 2).

The LCQ-MC and PC-QOL questionnaires were used to evaluate the frequency and severity of coughing episodes, their impact on the sleep patterns of both patients and parents, and the overall quality of life in children. The LCQ-MC scale is validated for children aged 6‐14 years old and consists of 19 items, and the PC-QOL is validated for children aged 0‐14 years old and consists of 27 items; both assess physical, psychological, and social functioning domains. The total score is calculated by summing the scores from each domain. The higher the accumulated score, the better the quality of life and the less the detrimental effects of coughing on children.

### Statistical Analysis

Measurement data with normal distribution are presented as means and SDs, and between-group comparisons were conducted using an independent sample *t* test or ANOVA. Measurement data without normal distribution are expressed as median (IQR), and nonparametric tests were used for intergroup comparisons. Count data are expressed as percentages, and chi-square tests were used for intergroup comparisons. Normally distributed variables were analyzed using Pearson correlation analysis, whereas variables without normal distribution were subjected to Spearman correlation analysis. Difference analysis and multivariate linear regression analysis were used to investigate the factors affecting the cough duration prior to the diagnosis in children. *P* values obtained from univariate comparisons were adjusted for multiple testing using the Benjamini-Hochberg false discovery rate (FDR) correction method, with a significance threshold set at Q<.05. Statistical analysis was conducted using IBM SPSS 26.0 software, with a *P* value of less than .05 exhibiting statistical significance. The FDR correction was carried out using the R statistical programming language (version 4.5.1; R Core Team).

### Ethical Considerations

This study was approved by the Ethics Committee of Qilu Hospital, Shandong University, under the ethical number KYLL-202111-118-1. Written informed consent was meticulously obtained from all participating children or their guardians. All data are anonymous.

## Results

### Sample Characteristics

As of November 2022, we enrolled 88 patients with PBB. Among these 88 enrolled children, there was a male predominance, with a male-to-female ratio of 1.84:1 and a median age of 60 (IQR 48‐78) months. The median cough duration prior to inclusion in this study was 8 (IQR 4.5‐16) weeks. Overall, 38% (33/88) of patients lived in rural areas, while 63% (55/88) of patients lived in urban areas. Furthermore, 51% (45/88) of patients had siblings and 49% (43/88) were only children. Parental education levels indicated that 28% (25/88) of patients had a high school education or below. In contrast, 72% (63/88) of patients had postsecondary education. The cohort analysis further demonstrated that 24% (21/88) of children were primarily cared for by their grandparents, while 76% (67/88) of children received primary care from their parents. Environmental tobacco smoke exposure was reported in 16% (14/88) of children, contrasting with 84% (74/88) of children without such exposure. Furthermore, regarding activity patterns during illness, 63% (55/88) of children continued attending school and physical activities despite cough symptoms, whereas 38% (33/88) rested at home. Parents’ assessment of the nature of the cough indicated that 16% (14/88) of parents mistook a wet cough (a cough producing phlegm) for a dry cough. Meanwhile, 84% (74/88) of parents correctly identified it.

All 88 children had prior outpatient visits at external health care facilities before enrollment in this study. Health care–seeking behavior analysis showed that 49% (43/88) of children visited a municipal-level or higher hospital, 13% (11/88) of children had more than 5 repeated visits. Diagnostic accuracy assessment revealed that 33% (29/88) of cases were initially misdiagnosed with cough variant asthma or bronchial asthma, and 65% (57/88) of children were incorrectly diagnosed with bronchitis or bronchopneumonia and were treated with antibiotics and Chinese patent medicine before study participation (Table S1 in Multimedia Appendix 3).

Complete pre-enrollment medication records were unavailable for some participants in this study. Of the 73 children with documented antibiotic use, 48 (66%) received β-lactam antibiotics (amoxicillin, clavulanate potassium, cefaclor, etc), with only 4 (5%) having treatment durations longer than 2 weeks. Additionally, 33 (45%) children were treated with macrolides, including azithromycin and erythromycin; among them, only 5 (7%) had a treatment duration exceeding 2 weeks (Table S2 in Multimedia Appendix 3).

As for microbiological findings, following enrollment in this study, 63 pediatric patients were hospitalized for treatment and underwent bronchoscopy and bronchoalveolar lavage. Pathogen tests, such as bronchoalveolar lavage fluid culture, polymerase chain reaction, and metagenomic next-generation sequencing analysis, were also conducted. Of the 63 patients, 39 (62%) were positive for pathogenic bacteria. Of the 39 patients, 17 *Streptococcus pneumoniae* (44%), 7 *Haemophilus influenzae* (18%), and 6 *Staphylococcus aureus* (15%) were the most frequently detected pathogens. Additionally, 36% (18/39) of patients were coinfected with viral infections. Rhinovirus had the highest detection rate among viruses. Fungi were detected in 2 patients, namely Candida albicans; Pseudomonas aeruginosa, a bacterium, was also identified (Table S3 in Multimedia Appendix 3).

All the children were prescribed oral antibiotic therapy after enrollment in this study. Forty-seven patients were treated with Cefditoren Pivoxil Granules (3 mg/kg, 3 times a day), with a mean treatment duration of 2.7 (SD 0.7) weeks; 27 patients received Faropenem Sodium Tablets (5 mg/kg, 3 times a day), with a mean duration of 3.0 (SD 1.1) weeks; and 22 patients were administered Linezolid Tablets (10 mg/kg, 3 times a day), with a mean treatment course of 2.5 (SD 1.3) weeks. Among these cases, 2 patients who showed inadequate response to Cefditoren Pivoxil Granules achieved cough resolution after switching to Faropenem Sodium Tablets; 4 patients who failed initial Cefditoren Pivoxil Granules therapy successfully transitioned to Linezolid Tablets with subsequent symptom disappearance; 2 patients received combination therapy with Faropenem Sodium Tablets and Linezolid Tablets, and 1 patient required antifungal medication. The median duration of anti-infective therapy after evaluation at our institution was 3 (IQR 2‐4) weeks. Treatment duration distribution was as follows: 31% (27/88) of children received 2 weeks of therapy, 33% (29/88) completed 3 weeks, 27% (24/88) required 4 weeks, and 9% (8/88) needed extended therapy beyond 4 weeks. Complete resolution of cough symptoms was achieved in all cases (Tables S4 and S5 in Multimedia Appendix 3).

### Analysis of Factors Affecting Cough Duration Prior to the Diagnosis

Ten potential influencing factors were screened through univariate analysis. Given that multiple comparisons increase the risk of Type I error, we applied the Benjamini-Hochberg method to control the FDR by adjusting the *P* values. A Q value <.05 after correction was considered statistically significant. The corrected results demonstrated statistically significant differences among groups for the following 4 factors: place of residence (*z*=−2.72; *P*=.006; Q=.045), parental education level (*z*=−2.37; *P*=.02; Q=.045), parental assessment of the nature of the cough (*z*=−2.46; *P*=.01; Q=.045), and whether the individual rested during the cough episode (*z*=−2.50; *P*=.01; Q=.045). In contrast, no significant differences were observed for other variables; detailed data are presented in Table 1.

**Table 1.** Analysis of the factors affecting the cough duration prior to the diagnosis (N=88).^a^

Variable and Group	Duration of cough (weeks), median (IQR)	*z* score	*P* value	Q value
Sex		−0.25	.81	.896
Male	8 (6‐14)			
Female	8 (4‐16)			
Residence		−2.72	.006	.045
City	8 (4‐12)			
Village	14 (8-26)			
Siblings		−1.92	.06	.110
Yes	12 (8-16)			
No	8 (4‐12)			
Parental education level		−2.37	.02	.045
Postsecondary education	8 (4‐12)			
High school education or below	12 (8-24)			
Judgment of the nature of cough		−2.46	.01	.045
Yes	8 (4‐12)			
No	14 (8-36)			
Rest at disease period		−2.50	.01	.045
Yes	8 (4‐12)			
No	12 (8-16)			
Primary caregiver		−0.07	.94	.944
Parents	8 (4‐16)			
Grandparents	8 (5‐14)			
Environmental exposure to smoking		−0.26	.79	.896
Yes	8 (4‐16)			
No	8 (5.5‐16)			
Treatment of asthma		−1.66	.10	.139
Yes	12 (8-20)			
No	8 (4‐12)			
Treatment with antibiotics		−1.71	.09	.139
Yes	12 (4-16)			
No	8 (6‐8)			

a The nonparametric test was conducted with the duration of cough prior to the diagnosis as the dependent variable.

To explore the independent effects of each variable on cough duration prior to the diagnosis after controlling for other factors, the 4 variables that showed significant associations (*P*<.05) in the univariate analysis (including residence, parental education level, parental assessment of the nature of the cough, and rest status at disease period) were included in a multivariate linear regression analysis model. The assignment of each variable is presented in Table 2. Specifically, place of residence (B=9.35, 95% CI 0.36-18.35; *P*=.04) and rest status during the coughing episode (B=7.87, 95% CI 0.36-15.38; *P*=.04) were significantly associated with cough duration prior to the diagnosis. In contrast, parental educational level (B=2.83, 95% CI −6.36 to 12.01; *P*=.54) and parental assessment of the nature of the cough (B=7.39, 95% CI −2.59 to 17.38; *P*=.15) were not significantly associated with the outcome. The analysis revealed that the regression model was statistically significant (*F*_4,83_=3.98; *P*=.005). The 4 predictor variables collectively explained 12.1% of the variance in the interval between symptom onset and diagnosis (adjusted *R*²=0.121) (Table 2).

**Table 2.** The factors affecting cough duration in multivariate linear regression analysis (N=88).^a^

Independent variable	B (SE)^b^		Standardized β coefficient	*t* value (*df*)	*P* value	VIF^c^
Constant	5.75 (3.26)		—^d^	1.77 (83)	.08	—
Residence	9.35 (4.52)		.24	0.07 (83)	.04	1.30
Education level of parents	2.83 (4.62)		.07	0.61 (83)	.54	1.32
Judgment of the nature of cough	7.39 (5.02)		.15	1.47 (83)	.15	1.03
Rest at disease period	7.87 (3.78)		.21	2.08 (83)	.04	1.02

a Variable description: Residence (city=0, village=1); education level of parents (postsecondary education=0, high school education or below=1); judgment of the nature of cough (correct=0, wrong=1); rest at disease period (rest=0, no rest=1)

b *R*2=0.161; adjusted *R*2=0.121; *F*_1_-score=3.98; *P*=.005; D-W value=1.81.

c VIF: variance inflation factor.

d Not available.

### Correct Antibiotics Significantly Reduced Daytime and Nocturnal Cough Scores

At the first visit, all 88 children were scored by their parents based on the symptoms. At the first visit, 5% (4/88) of patients only had night cough, 23% (20/88) of patients only had daytime cough, and 20% (18/88) of patients had frequent daytime cough affecting their daytime life. There were 32% (28/88) patients with frequent nocturnal cough affecting sleep, and 13% (11/88) patients with frequent daytime and nocturnal cough affecting daily life (Table S6 in Multimedia Appendix 3). At the first visit, there was no significant difference in daytime cough symptom score and nocturnal cough symptom score between the two groups (*z*=−0.52; *P*=.60; Table S7 in Multimedia Appendix 3).

The daytime and nocturnal scores at 2 weeks and 4 weeks after diagnosis were assessed via outpatient examination and telephone follow-up. Fortunately, all parents and caregivers of the enrolled patients completed the assessment of their children’s cough symptom scores at both the 2nd and 4th weeks of treatment. At the 2nd week of follow-up, daytime cough disappeared in 38% (33/88) of patients, nocturnal cough disappeared in 70% (62/88) of patients, and the cough symptoms completely disappeared in 31% (27/88) of patients. At the 4th week of follow-up, daytime cough symptoms disappeared in 90% (79/88) of patients, night cough symptoms disappeared in 98% (86/88) of patients, and cough symptoms completely disappeared in 90% (79/88) of patients (Tables S8 and S9 in Multimedia Appendix 3).

Daytime cough did not completely disappear in 9 patients after 4 weeks of treatment. Cough disappeared in 7 patients after 5 to 6 weeks of antibiotic therapy, aggravated in 1 patient and improved after adding antifungal drugs in the hospital, and 1 child did not continue treatment. The nocturnal cough did not completely disappear in 2 children at 4 weeks of treatment, and the duration of treatment was prolonged to 5 weeks (Tables S8 and S9 in Multimedia Appendix 3).

### PBB Reduced the Quality of Life in Children

In total, 35 PBB children older than 6 years old completed the LCQ-MC. The results showed no significant difference in the physical, psychological, and social scores of LCQ-MC among children with PBB (*F*_2,102_=0.13; *P*=.88; Table S10 in Multimedia Appendix 3 and Table 3).

**Table 3.** Score of the Leicester Cough Questionnaire in Mandarin-Chinese (LCQ-MC) and Parent-Proxy Cough-Specific Quality of Life (PC-QOL).

Scale and domain	Score, mean (SD)	*F* test (*df*)	*P* value
LCQ-MC (n=35)		0.13 (2,102)	.88
Physical	4.90 (0.89)		
Psychological	4.99 (1.28)		
Social	4.85 (1.37)		
PC-QOL (n=88)		3.20 (2,261)	.04
Physical	3.10 (1.36)^a^		
Psychological	3.32 (1.57)^a^^,^^b^		
Social	3.67 (1.53)^b^		

a Data were analyzed by repeated-measures ANOVA followed by Bonferroni post hoc test. Within each scale, different superscript letters denote statistically significant differences at *P*<.05 in the post hoc pairwise comparisons.

b Data were analyzed by repeated-measures ANOVA followed by Bonferroni post hoc test. Within each scale, different superscript letters denote statistically significant differences at *P*<.05 in the post hoc pairwise comparisons.

The reliability and validity of the PC-QOL scale were measured among 88 children at the first visit. Cronbach α coefficient was higher than 0.8, suggesting good internal consistency and high reliability. Cronbach α coefficients of physical, psychological, and social components were all higher than 0.8, suggesting that the scale had high reliability. Kaiser-Meyer-Olkin (KMO) >0.8 was suitable for factor analysis. The overall KMO of PC-QOL was 0.90, and the KMOs of physical, psychological, and social components were all >0.8, suggesting that the scale exhibited good construct validity (Table S11 in Multimedia Appendix 3). The results showed a significant difference between the scores of the physical domain and social domain among children with PBB (*t*_174_=−2.58, *P*=.01; Table 3), and the score of the physical domain was significantly lower than that of the social domain.

In total, 19 parents of children with PBB completed the PC-QOL scale at baseline and after treatment. The results showed significant differences in the overall distribution of each domain and the total score of PC-QOL between baseline and after treatment (Table 4). The scores of physical (*t*_18_=−6.05, *P*<.001), psychological (*t*_18_=−4.42, *P*<.001), and social domains (*t*_18_=−4.79, *P*<.001) and total scores (*t*_18_=−5.25, *P*<.001) after treatment were significantly higher than those before treatment.

**Table 4.** Parent-Proxy Cough-Specific Quality of Life scores were compared before and after treatment using the paired samples *t* test (n=19).

Domain	Before treatment, mean (SD)	After treatment, mean (SD)	*t* test (df)	*P* value
Physical	2.86 (1.35)	5.48 (1.24)	−6.05 (18)	<.001
Psychological	3.38 (1.54)	5.69 (1.17)	−4.42 (18)	<.001
Social	3.63 (1.60)	5.89 (1.06)	-4.79 (18)	<.001
Sum	9.87 (4.26)	17.06 (3.22)	-5.25 (18)	<.001

Thirty-five children with PBB aged more than 6 years were included in this study. Pearson correlation analysis and difference analysis showed a good correlation between the scores and total scores of PC-QOL and LCQ-MC (physical: *r*=0.57, *P*<.001; psychological: *r*=0.48, *P*=.004; social: *r*=0.58, *P*<.001; total: *r*=0.66, *P*<.001) (Table S12 in Multimedia Appendix 3). The scores and total scores of PC-QOL were significantly lower than those of LCQ-MC (physical: *t*_34_=8.31, *P*<.001; psychological: *t*_34_=6.58, *P*<.001; social: *t*_34_=5.09, *P*<.001; total: *t*_34_=8.11, *P*<.001) (Table 5).

**Table 5.** Paired sample *t* test was used for the Leicester Cough Questionnaire in Mandarin-Chinese (LCQ-MC) and Parent-Proxy Cough-Specific Quality of Life (PC-QOL) among children aged ≥6 years (n=35).

Domain	LCQ-MC, mean (SD)	PC-QOL, mean (SD)	*t* test (df)	*P* value
Physical	4.90 (0.89)	3.24 (1.43)	8.31 (34)	<.001
Psychological	4.99 (1.28)	3.38 (1.53)	6.58 (34)	<.001
Social	4.85 (1.37)	3.73 (1.49)	5.09 (34)	<.001
Sum	14.74 (3.11)	10.35 (4.21)	8.11 (34)	<.001

## Discussion

### Principal Findings

This study examined factors contributing to the prolonged clinical course of PBB and evaluated the impact of chronic cough on quality of life in children with PBB. Using multiple regression analysis, this study identified place of residence and whether the child was resting or active during coughing episodes as significantly associated with prediagnosis cough duration. Furthermore, PBB substantially impairs multiple dimensions of children’s well-being, including physical, psychological, and social functioning.

### Analysis of Factors Affecting Cough Duration Prior to the Diagnosis

PBB is a common cause of chronic wet cough among preschool children aged 0‐6 years [26,27]. The 2019 multicenter study on the proportion of the causes of chronic wet cough in children [28] showed that PBB was the 4th cause of chronic wet cough among children in China. It was also the leading cause of chronic wet cough among children younger than 1 year. Early diagnosis and treatment of PBB can reduce the duration of persistent cough, improve clinical symptoms, prevent its progression to bronchiectasis, and reduce morbidity and mortality [27,29].

Residence in rural areas demonstrated the strongest correlation with prolonged cough duration prior to PBB diagnosis, underscoring a core issue of inequitable distribution of medical resources. Compared to urban residents, rural families likely face several barriers, including limited access to specialized diagnostic facilities and expertise. PBB was recognized as a diagnostic name worldwide in 2008 [30]. In 2013, PBB was first included in the guidelines for chronic cough in China [4]. However, the Australian scholar Laird et al [31] conducted a survey of local medical workers in towns and remote communities and found that approximately one-third of local medical workers did not know the guidelines for chronic wet cough and had little knowledge about chronic wet cough, PBB, and chronic suppurative lung disease. They normalized chronic wet cough. The long-term complications of chronic wet cough and the need for timely assessment and therapeutic intervention have been largely neglected. Therefore, the cough duration of children with PBB who live in rural areas is longer than that of those living in urban areas, which is closely associated with the lack of knowledge about PBB in primary medical institutions in rural areas of China. Residents in rural areas may experience significant delays within the local health care system. They often undergo multiple rounds of ineffective empirical treatments at primary care facilities before receiving a definitive diagnosis. A study including 190 children with PBB diagnosed after referral to secondary care institutions [32] reported that more than 80% of the children had visited a doctor 5 or more times in the past 12 months, and 53% (101/190) had visited a doctor 10 or more times before referral. Therefore, there is a lack of awareness of PBB-related treatment options in primary care institutions. Geographical barriers: accessing medical centers that provide specialized pediatric respiratory care requires considerable financial resources and time; this poses a substantial barrier to timely diagnosis. The family in rural residents had insufficient awareness of disease prevention and control and had less knowledge about chronic diseases of the respiratory system. Due to the economic conditions of families, parents more often choose the nearby diagnosis and treatment facilities or buy drugs based on their choice rather than going to professional medical institutions for treatment. When the child’s condition does not improve, they will seek more professional treatment. In addition, parents in rural areas have less energy to take care of their children, which limits the implementation of medical advice and affects the treatment effect. Perhaps in the future, we will be able to introduce advanced technology such as cough sound classifiers [33], eHealth systems and interventions [34,35] to offer a promising solution for the early detection and efficient management of PBB in primary care institutions.

Prior to the diagnosis of PBB, inadequate rest during the coughing episode was significantly linked to a prolonged duration of the cough. In this study, 63% (55/88) of the children continuously went to school after the onset of cough symptoms. Among daycare children, those who receive care because of a cough are 13 times more likely to develop a chronic cough than those who stop caring for children at home [36]. The incidence of infectious diseases among children in daycare or preschool education is 2 to 3 times that of ordinary children [37,38], and the large population density in the classroom can easily lead to cross-infection of viruses among children. Common respiratory pathogens, such as respiratory syncytial virus, influenza A virus, and rhinovirus, upregulate the bacterial adhesion molecules of epithelial cells in children, which can also lead to mucociliary clearance disorders and damage epithelial cells, affect the clearance efficiency, promote bacterial invasion of host cells and tissues, and enhance bacterial growth and colonization. This damage can take weeks to repair [39-41]. Cross-infection provides insufficient time to repair the airway mucosa, and repeated infection worsens airway inflammation, further exacerbating airway inflammation, thus forming a vicious circle, which is conducive to the formation of bacterial biofilm [42], promotes the development and progression of PBB, and prolongs cough symptoms. However, due to work requirements and social and family tasks, it is difficult in many families in our country for parents to terminate school and nursery care for children with a persistent cough. In contemporary society, seeking medical care for children often entails practical constraints such as taking time off work or arranging leave from school. Consequently, families tend to prioritize health care visits only when symptoms become sufficiently severe to cause significant functional impairment, that is, when the child must rest and daily routines are substantially disrupted.

Parents play critical roles in the treatment of children with respiratory diseases [43]. Studies have found that the low education level of primary caregivers is a risk factor for the incidence of recurrent upper respiratory tract infections among children [44,45]. A low education level is a risk factor for the low awareness of respiratory infection prevention and control among parents of preschool children [46]. At the same time, in the process of treatment, parents with higher education levels will provide accurate information, have better compliance, and will not arbitrarily stop the drug. Nevertheless, parental education showed no significant association with diagnostic delay in the multivariate model. One possible explanation is that parental education influences the disease course indirectly through various health behaviors and environmental factors. Research shows that a mother’s education level is a factor influencing children’s susceptibility to illnesses [47-49]. Families with higher levels of parental education are more likely to seek professional medical care when their children are ill, possibly because higher education enhances health knowledge and the ability to access health care resources. This influence may be reflected in variables such as urban versus rural residence and the frequency or severity of cough symptoms. When variables such as residence and cough characteristics are included in statistical models, the independent effect of parental education becomes attenuated or statistically insignificant.

Moreover, this study indicated that parental judgment of the nature of the cough was not significantly associated with the duration of cough prior to diagnosis. Like parental education level, parental judgment may be influenced by mediating effects and potential collinearity with other variables. These factors could reduce its independent explanatory power. Furthermore, this result suggests that even when parents are able to recognize key symptoms such as “wet cough,” this recognition does not necessarily translate into timely health-seeking behavior. Foreign studies have shown that local Australian residents believe that isolated chronic wet cough does not need medical management [50], which often delays treatment. Some parents may also rely on self-management approaches based on previous illness experiences. Specifically, a productive cough may be mistaken for a mild condition that resolves on its own, such as the recovery phase of a common cold, rather than being recognized as a symptom that requires professional medical evaluation.

### Quality of Life Score Among Children With PBB

Frequent cough during the daytime can affect children’s learning and life, and nocturnal cough can affect children’s sleep, lower daytime energy levels, and affect children’s health. Long-term cough can also lead to anxiety, embarrassment, and other psychological problems, and repeated medical treatment and physical discomfort can also affect the progress of school life. Chronic cough affects children’s physical, sleep, and mental health and school performance. It can also cause anxiety in parents, interfere with work and other plans, and increase family expenditure. The decreased quality of life caused by cough may be the direct cause of patients’ visits.

The LCQ scale [20] was first proposed by SSB in 2003, which assesses the effects of chronic cough on 19 items across 3 domains: physical, psychological, and social domains. The LCQ scale is highly reproducible and responsive to change. It is a reliable and valid measurement tool for assessing cough-specific health status and can be used to evaluate the effects of cough on different aspects over time, with a minimal clinically important difference of 1.3 [51]. Gao et al [25] translated the LCQ scale into Mandarin Chinese in 2014 using the forward-backward translation method, creating the LCQ-MC. There were no significant differences in the conceptual content between the back-translation and the original English version. This was verified by S.S. Birring (SSB), the author of the English LCQ. This study confirmed that the LCQ-MC effectively assessed the severity of cough symptoms in patients with bronchiectasis. Research by Xu et al [52] indicated that the LCQ-MC can assess cough severity after thoracoscopic surgery in patients with pulmonary disease. The Chinese National Guideline on Diagnosis and Management of Cough (2021) [53] also recommends using LCQ-MC to assess cough-related quality of life, supporting these findings.

The PC-QOL scale [21] was developed by Newcombe PA in 2008. The questionnaire was set according to the opinions of experts in child respiratory medicine and psychology. It comprises 27 items involving physical, psychological, and social aspects. The parents’ feelings (15 items) and concerns (12 items) about the severity of cough in children with chronic cough were evaluated to reflect the effect of children’s cough on parents and parents’ perception of their children’s quality of life. In 2010, Newcombe et al [54] confirmed that the questionnaire was a reliable and valid measure, with a minimally important difference of 0.9 [55], capable of assessing childhood cough-related quality of life over specified periods. But over the last 10 years, minimal research has been conducted on the creation, application, and cultural modification of QOL evaluation instruments for chronic cough in children [56].

The scores of 35 children who completed both LCQ-MC and PC-QOL at the first visit were analyzed to explore the consistency of quality of life assessment between children and their parents. Correlation analysis showed a good correlation between PC-QOL and LCQ-MC in physical, psychological, and social domains and total scores. After cough remission, the scores of each domain and the total score of the PC-QOL scale significantly increased in children with PBB, suggesting that the scale has good therapeutic responsiveness and can be used to assess treatment effects. A randomized controlled trial [57] also showed that the PC-QOL score of children with PBB increased by 2.1 and 1.9 after 2 or 4 weeks of treatment with amoxicillin clavulanate potassium, respectively. A study [58] also indicated that PC-QoL scores were significantly improved postintervention. The improvement in the PC-QOL score suggests an improvement in the quality of life of children and their caregivers. The pressure and concerns of parents will disappear when the children’s cough stops [32]. The treatment of PBB not only relieves the cough symptoms of children and solves the physical discomfort of children but also significantly improves the psychological and social effects of cough on children and their caregivers.

In this study, the average scores of the physical, psychological, and social domains of the LCQ-MC scale were mean 4.90 (SD 0.89), mean 4.99 (SD 1.28), and mean 4.850 (SD 1.37), respectively, and the average total score of each domain was mean 14.74 (SD 3.11). In the study conducted by Reynolds et al [59], the average scores of physical, psychological, and social domains of LCQ were mean 6.53 (SD 0.39), mean 6.83 (SD 0.34), and mean 6.87 (SD 0.27), respectively. The total scores of LCQ ranged from 17.05 to 21, with an average score of mean 20.23 (SD 0.85). The study suggested the following scores as the lower limits of a normal LCQ score: physical domain: 5.36; psychological domain: 5.81; social domain: 6.06; and total score: 17.68. In our study, the scores of each domain and the total score of the LCQ-MC scale among children with PBB were lower than the lower limit of normal scores suggested by the above studies, suggesting that PBB can decrease the physical, psychological, and social quality of life in children.

The scores of PC-QOL were significantly lower than those of LCQ-MC. The differences in scores between PC-QOL and LCQ-MC could be attributed to the characteristics of parents and children in families. Parents’ response is affected by their expectations and attention to their children, the burden of care, their own mental health, and family responsibility [60]. Children’s quality of life score was higher than that of parents’ evaluation. Consistently, previous studies have found that the self-rated quality of life scores of children with attention-deficit hyperactivity disorder syndrome, sickle cell anemia [61], and ataxia telangiectasia [62] were higher than those of their parents.

On the LCQ-MC scale, the lowest score was observed in the social domain, and the highest score was observed in the psychological domain. However, on the PC-QOL scale, the lowest score was observed in the physical domain and the highest score was observed in the social domain. Parents are more concerned about the effects of cough on the body of children, while the social function of children is more affected, suggesting that parents of children with PBB may ignore the effect of chronic cough on the social function of children. For children, it is critical to achieve good peer relationships, and children with chronic diseases more frequently report that their health problems impair their participation in group activities and affect their quality of life [63-65]. For children with PBB, chronic cough is often considered a sign of being unhealthy and infected. Children themselves worry about the effect of cough on peers, care about the views of classmates and teachers, and are unwilling to go to school. In addition, frequent absences in school and group activities due to repeated medical visits and exacerbation of cough symptoms subsequent to respiratory tract infection also limit the time of children to participate in social and recreational activities, thus decreasing their social communication skills. At the same time, the parent-child relationship between the children and their parents will also be affected, and the parents’ requirements, control, and overprotection of the children will increase, and parents of the children will have to complete work tasks to earn sufficient money for taking care of their children, which can induce a negative feeling. Negative attitudes toward children can affect communication with children, and some parents even give their children to others because they cannot cope with their children’s diseases, which affects children’s psychosocial function [66]. Based on the bio-psycho-social medical model, people’s health not only represents their normal physical function, but also the health and harmony of physiological, psychological, and social functions. Medical workers should not only solve the physical pain of patients but also improve their quality of life.

Therefore, clinicians should pay more attention to children with PBB who live in rural areas and whose parents have a low education level. Parents should consider the rest time at home for children with PBB to shorten the duration of the cough. PBB can seriously affect the physical, mental, and social functions of children. While treating the physical diseases of children, doctors and parents should also pay attention to the detrimental effects of cough on the psychological and social functions of children and strengthen psychological counseling and communication to help children improve their quality of life.

### Limitations

Of course, this study has several limitations. First, the adjusted *R*² value of 0.121 suggests that a considerable proportion of the variance in cough duration remains unexplained. This indicates the potential role of unmeasured confounding factors, such as specific pathogens, the child’s atopic predisposition, and variations in both the accuracy of primary care diagnoses and the level of awareness among primary care providers. Additionally, only Chinese participants were included in this study. No analysis of cultural or social influences related to racial or ethnic differences was conducted. Second, all measures, including symptom data and clinical assessments, relied on subjective information, which is susceptible to measurement bias, such as recall or reporting bias. Third, the relatively small sample size may limit the statistical power of the analyses, and the cross-sectional design precludes causal inference between variables. As a result, the findings are preliminary. Future studies should use longitudinal designs and recruit larger samples. They should also incorporate laboratory-confirmed bacterial diagnoses and environmental assessments to validate these findings and better elucidate the biological and sociobehavioral mechanisms influencing the course of PBB in children.

### Conclusions

Based on univariate analysis, 4 variables potentially associated with cough duration prior to PBB diagnosis were identified and included in the multivariate regression model. The final model revealed that “place of residence” and “rest status during the coughing episode” were significantly associated with cough duration. Nevertheless, the model also indicated that a substantial proportion of the variance remains unexplained, suggesting the involvement of additional unmeasured factors that warrant further investigation. Moreover, our findings indicate that PBB significantly impairs children’s physical, psychological, and social quality of life. Notably, changes in social functioning are often overlooked by both clinicians and parents during the diagnosis, treatment, and daily care of affected children.

## Supplementary material

10.2196/82887 Multimedia Appendix 1 Patients’ demographics assessments with a case report form.

10.2196/82887 Multimedia Appendix 2 Questionnaires of simplified cough symptom score, cough symptom score, Leicester Cough Questionnaire, Leicester Cough Questionnaire in Mandarin-Chinese, and Parent-Proxy Cough-Specific Quality of Life.

10.2196/82887 Multimedia Appendix 3 Characteristics of 88 children, usage of antibiotics outside the hospital, pathogen detection in the bronchoalveolar lavage fluid, antibiotic use, duration of antimicrobial therapy, sCSS at first visit, comparison of CSS between day and night, efficacy evaluation of daytime cough, efficacy evaluation of nocturnal cough, LCQ-MC score, the reliability and validity of PC-QOL, and correlation analysis of LCQ-MC and PC-QOL scores among children aged ≥6 years. LCQ-MC: Leicester Cough Questionnaire in Mandarin-Chinese; PC-QOL: Parent-Proxy Cough-Specific Quality of Life; sCSS: simplified Cough Symptom Score.

## Abbreviations

KMO: Kaiser-Meyer-Olkin
LCQ: Leicester Cough Questionnaire
LCQ-MC: Leicester Cough Questionnaire in Mandarin-Chinese
PBB: protracted bacterial bronchitis
PC-QOL: Parent-Proxy Cough-Specific Quality of Life
sCSS: simplified Cough Symptom Score
