# Supplementary material for: Analysis of Cough Factors and Quality of Life Score Among Children With Protracted Bacterial Bronchitis: Cross-Sectional Study
Source: JMIR Pediatr Parent. 2025 Dec 19;8:e82887. doi: 10.2196/82887 (PMC12716831; doi:10.2196/82887)
Supplement: Multimedia Appendix 1 [file pediatrics-v8-e82887-s001.pdf]

Analysis of Prolonged Course in Children with Chronic Cough

(Qilu Hospital of Shandong University)

Case Report Form (CRF)

Initial: |__|__|__|__|

Patient ID: |__|__|__|__| __|

CRF Filler:________________

CRF Completion Date: |__|__|__|__| |__|__| |__|__|

Instructions for Completion

1.Please complete the form in ink, ensuring legible and clearly distinguishable handwriting.

2.Subject name format (Chinese Pinyin initials):

- Two-character names: Enter the first two letters of each character (left-aligned).
- Three-character names: Enter the initial of each of the first two characters, followed by the initial and second letter of the third character.
- Four-character names: Enter the initial of each character.

Examples：

Zhang Hong |_Z|_H|_H|_O| Yang Shuli |_Y|_S|_L|_I|

Shangguan Haoqi |_S|_G|_H|_Q|

3.Selection Criteria:

- Mark the appropriate option with "√" in all checkboxes (¨).
- All fields in the form must be completed with relevant text or numerical entries; blank entries are not permitted.

4. Data Correction Protocol:

- All entries must be accurate and free from unauthorized modifications. If an error is identified:
- Draw a single horizontal line through the incorrect entry.
- Enter the correct information adjacent to the original entry.
- Clearly annotate with the corrector's initials (capitalized), followed by the modification date.
- Example: If the correct value should be 67.9 but was erroneously recorded as 75.4, the correction should appear as:~75.4~ → 67.9 [NHN] 2021-11-13
- Prohibited Actions: Do not obscure original records using erasers, correction fluid, or any other means.

5.Missing Data Handling:

- For unspecified drug dosages or administration times, enter "NK" (Not Known) to explicitly indicate unavailable information.

6.Case Report Form (CRF) Integrity:

- Do not alter the original format of the CRF. If insufficient space is available for required documentation:

- Record the additional information on a dedicated supplemental page appended to the CRF.
- Retain an identical copy of all supplementary records for archival purposes.

7.Informed Consent Documentation:

- The informed consent form must be signed by the study participant (patient) personally.
- Exception: In cases where the participant lacks legal capacity or exhibits impaired decision-making ability, the legally authorized representative (LAR) may provide signature on behalf of the participant.

Patient Demographic Information

Name：   Gender：   Date of Birth：   Age：

Medical Record Number：

Primary Contact Address： ____________________________________

Next of Kin： ____________   Relationship to Patient:： _____________

Mobile Phone Number： _________________

Landline Telephone Number: __________________________

Birth and Family History

Preterm Birth<br>☐Y ☐N	Low Birth Weight<br>☐Y ☐N	ICU Management ☐Y ☐N<br>Reason：☐☐☐☐☐☐☐☐☐☐
Residential Address ☐Rural ☐Urban		
Residential Address ☐3 ☐4 ☐5 ☐6 ☐7 ☐8 ☐>8		
Sibling Count ☐1 ☐2 ☐3 ☐4 ☐>4		
Birth Order Among Siblings ☐1 ☐2 ☐3 ☐4 ☐5		
Paternal Age<br>☐☐	Paternal Occupation:	Educational Attainment: ☐Junior high school or below ☐High school ☐ vocational college ☐Bachelor's degree ☐Master's degree ☐Doctoral degree or higher
Maternal Age<br>☐☐	Maternal Occupation :	Educational Attainment: ☐Junior high school or below ☐High school ☐ vocational college ☐Bachelor's degree ☐Master's degree ☐Doctoral degree or higher

Living Conditions of Pediatric Patients

Dietary Habits

☐ Balanced diet (varied intake) ☐ Primarily meat, eggs and dairy-based ☐ Primarily carbohydrate-based (e.g. rice/noodles) ☐ Primarily vegetable and fruit-based ☐ Low in vegetables and fruits

Use of Dietary Supplements

☐ None ☐ Vitamins A/B/C/D ☐ Micronutrients (e.g., zinc, iron) ☐ Probiotics
☐ Other (specify: ______)

Perceived Academic Stress

☐ Minimal ☐ Mild ☐ Moderate ☐ Significant ☐ Severe ☐ Extreme

Average daily outdoor activity time in spring and autumn (hours):

Average daily outdoor activity time in winter (hours):

Average daily outdoor activity time in summer (hours):

Primary Caregiver(s)

☐ Parents ☐ Grandparents ☐ Hired nanny/domestic helper

Whether the person accompanying the child to medical visits is the primary caregiver

☐N ☐Y

Past Medical and Family History

Past Medical History

☐ Congenital heart defect (CHD) ☐ Myocarditis ☐ Arrhythmia ☐ Hypertrophic cardiomyopathy ☐ Malnutrition ☐ Anemia ☐ Hematologic malignancies (e.g., leukemia) ☐ Immunodeficiency ☐ Ear diseases ☐ Allergic rhinitis ☐ Adenoid hypertrophy ☐ History of wheezing or asthma ☐ Gastrointestinal diseases
☐ Immunosuppressant use ☐ None

Vaccination History

Pertussis Vaccine (Whooping Cough Vaccine) ☐Yes ☐No

BCG Vaccine (Bacillus Calmette-Guérin Vaccine) ☐Yes ☐No

Allergic Manifestations

☐Cutaneous allergic manifestations ☐Allergic rhinitis ☐Allergic conjunctivitis ☐None

Allergen Testing Results

☐No allergens detected ☐Allergens detected : ☐☐☐☐☐☐☐☐☐☐☐☐☐☐

History of Exposure to Specific Substances

☐Mold ☐Tuberculosis (TB) ☐Livestock (cattle/sheep) ☐Smoke and irritants ☐Dust ☐Pets ☐None

Family History of Hereditary Disorders

☐ Negative ☐ Positive : ☐☐☐☐☐☐☐☐☐☐

History of Familial Infectious Diseases

☐ No ☐ Yes : ☐☐☐☐☐☐☐☐☐☐

Healthcare-Seeking Behaviors

☐ Self-management for minor illnesses (avoiding professional care) ☐ Pharmacist-guided medication purchase ☐ Self-diagnosis via online searches ☐ Telemedicine consultation
☐ Community health center/clinic ☐ Traditional Chinese Medicine (TCM) practitioner
☐ Non-respiratory medical specialists ☐ Respiratory specialists

Patient ID： |__|__|__|__|__| Initial： |__|__|__|__

Prodromal Symptoms and Cough Characteristics

**Fever**<br>☐ Present ☐ Absent	**Nasal Congestion**<br>☐ Present ☐ Absent	**Sneezing Attacks**<br>☐ Present ☐ Absent
**Nasal Discharge (Rhinorrhea)**<br>☐ Absent ☐ Present ☐ Clear mucus ☐ Yellow discharge ☐ White sticky mucus		
**Nausea**<br>☐ Present ☐ Absent	**Vomiting**<br>☐ Present ☐ Absent	**Foreign Body Aspiration**<br>☐ Present ☐ Absent
**Duration of Cough**<br>☐Days ☐ Months ☐ Years ☐Years and ☐Month		
**Timing of Cough Onset**<br>☐ Morning (upon waking) ☐ Daytime ☐ Midday ☐ Before bedtime<br>☐ Early morning (3-6 AM) ☐ After physical activity ☐ After cold air exposure<br>☐ After irritant exposure ☐ Postprandial (after meals) ☐ After oral medication		
**Daytime Cough Severity**<br>☐ None ☐ Occasional brief cough ☐ Frequent cough with minimal functional interference ☐ Frequent cough causing severe functional impairment<br>**Nighttime Cough Severity**<br>☐ None ☐ Occasional brief cough before/during sleep onset ☐ Frequent cough with mild sleep disturbance ☐ Frequent cough causing severe sleep disruption		
**Cough Characteristics and Sound**<br>☐ Dry/non-productive ☐ Wet/productive (with sputum) ☐ Throat-clearing type<br>☐ Barking/honking (croup-like) ☐ Brassy (metallic resonance) ☐ Stridor with whooping (paroxysmal inspiratory whoop)		

Associated Symptoms

**Fever**<br>☐Present☐ Absent	**Wheezing**<br>☐ Rhonchi (coarse crackles with secretions) ☐ Tachypnea (rapid shallow breathing) ☐ Stridor (monophonic inspiratory sound)<br>☐ Sibilant rhonchi (high-pitched whistling)		
**Sputum Production**<br>☐ Absent ☐ Present ☐ Yellow sputum ☐ White sticky sputum ☐ White frothy sputum ☐ Yellow-green purulent sputum			
**Nausea**<br>☐Present☐Absent	**Vomiting**<br>☐Present☐Absent	**Retrosternal Burning Sensation**<br>☐Present☐Absent	**Otalgia and Tinnitus**<br>☐Present☐Absent

Physical Examination by External Physicians

☐ None documented

☐ Documented

Conjunctival Congestion

☐ Present ☐ Absent ☐ Not documented

Nasal Examination Findings

☐ Mucosal congestion and swelling ☐ Thick/viscous secretions ☐ Normal examination
☐ Not documented

Pharyngeal Examination Findings

☐ Erythema/congestion ☐ Tonsillar hypertrophy ☐ Follicular hyperplasia
☐ Mucoid/purulent exudate coating ☐ Normal examination ☐ Not documented

Lung Auscultation Findings

☐Stridor (high-pitched inspiratory sound) ☐Wheezes (polyphonic expiratory whistling)
☐ Rhonchi (low-pitched coarse snoring) ☐ Gurgling sounds (secretions in large airways)
☐ Crackles/Rales (discontinuous bubbling) ☐ No adventitious sounds ☐ Not documented

External Hospital Test Results

Complete Blood Count (CBC)
☐ Not documented ☐ Within normal limits
☐ Abnormal results -Specify abnormalities:

CRP ☐ Not documented ☐ Within normal limits ☐ Abnormal -Specify value:

ESR ☐ Not documented ☐ Within normal limits ☐ Abnormal -Specify value:

PCT ☐ Not documented ☐ Within normal limits ☐ Abnormal -Specify value:

SAA ☐ Not documented ☐ Within normal limits ☐ Abnormal -Specify value:

T-SPOT ☐ Not documented ☐ Within normal limits ☐ Abnormal -Specify value:

MP-Ab ☐ Not documented ☐ Within normal limits ☐ Abnormal -Specify value:

IgE ☐ Not documented ☐ Within normal limits ☐ Abnormal -Specify value:

Cardiac Enzymes ☐ Not documented ☐ Within normal limits
☐ Abnormal -Specify value:

Troponin ☐ Not documented ☐ Within normal limits ☐ Abnormal -Specify value:

Cellular Immunity ☐ Not documented ☐ Within normal limits
☐ Abnormal -Specify value:

Humoral Immunity ☐ Not documented ☐ Within normal limits
☐ Abnormal -Specify value:

Inhaled & Food Allergen Screening ☐ Not documented ☐ Within normal limits
☐ Abnormal -Specify value:

Pulmonary Ventilation Function Parameters
☐ Not documented ☐ Within normal limits ☐ Abnormal
FEV1 measured/predicted (%) FEV1/Vcmax(%) FVC measured/predicted (%)
FVC measured/predicted (%) VT(ml/kg) TPTEF/TE TPTEF/VE

Bronchodilation Test (BDT)
☐ Not documented ☐ Within normal limits ☐ Abnormal -Improvement rate：

FeNO☐ ☐<br>☐Not documented	CaNO☐ ☐<br>☐Not documented	FnNO☐ ☐<br>☐Not documented

Lung Radiograph ☐ Not documented ☐ Within normal limits
☐ Abnormal -Specify value:

CT ☐ Not documented ☐ Within normal limits ☐ Abnormal -Specify value:

Laryngoscopy ☐ Not documented ☐ Within normal limits
☐ Abnormal -Specify value:

Bronchoscopy ☐ Not documented ☐ Within normal limits
☐ Abnormal -Specify value:

Electrocardiogram (ECG/EKG) ☐ Not documented ☐ Within normal limits
☐ Abnormal -Specify value:

Echocardiography ☐ Not documented ☐ Within normal limits
☐ Abnormal -Specify value:

Previous Treatments and Outcomes (Name, Type, Dosage, Duration, Outcome)

Medical Record 1

Hospital and Diagnosis:

Treatment Regimen and Efficacy:

☐ Antibiotics ☐ Nebulization therapy ☐ Mucolytics/Antitussives
☐ Leukotriene receptor antagonists
☐ Antihistamines ☐ Intranasal corticosteroids ☐ Mucolytic agents
☐ Traditional Chinese Medicine/Tuina/Physiotherapy

Medical Record 2

Hospital and Diagnosis:

Treatment Regimen and Efficacy:

☐ Antibiotics ☐ Nebulization therapy ☐ Mucolytics/Antitussives
☐ Leukotriene receptor antagonists
☐ Antihistamines ☐ Intranasal corticosteroids ☐ Mucolytic agents
☐ Traditional Chinese Medicine/Tuina/Physiotherapy

Medical Record 3

Hospital and Diagnosis:

Treatment Regimen and Efficacy:

☐ Antibiotics ☐ Nebulization therapy ☐ Mucolytics/Antitussives
☐ Leukotriene receptor antagonists
☐ Antihistamines ☐ Intranasal corticosteroids ☐ Mucolytic agents
☐ Traditional Chinese Medicine/Tuina/Physiotherapy

Medical Record 4

Hospital and Diagnosis:

Treatment Regimen and Efficacy:

☐ Antibiotics ☐ Nebulization therapy ☐ Mucolytics/Antitussives
☐ Leukotriene receptor antagonists
☐ Antihistamines ☐ Intranasal corticosteroids ☐ Mucolytic agents
☐ Traditional Chinese Medicine/Tuina/Physiotherapy

Rest Status

☐ Home rest ☐ Attending school as usual ☐ Physical exercise as rehabilitation
☐ Outdoor recreational activities

Current Clinical Condition

<table>
<tr><th colspan="4">Cough Characteristics and Associated Symptoms</th></tr>
<tr><td colspan="4">Timing of Cough Onset
☐ Morning (upon waking) ☐ Daytime ☐ Midday ☐ Before bedtime ☐ Early morning (3-6 AM) ☐ After physical activity ☐ After cold air exposure ☐ After irritant exposure ☐ Postprandial (after meals) ☐ After oral medication</td></tr>
<tr><td colspan="4">Cough Frequency
☐ Frequent ☐ Paroxysmal ☐Occasional</td></tr>
<tr><td colspan="4">Cough Characteristics and Sound
☐ Dry/non-productive ☐ Wet/productive (with sputum) ☐ Throat-clearing type
☐ Barking/honking (croup-like) ☐ Brassy (metallic resonance) ☐ Stridor with whooping (paroxysmal inspiratory whoop)</td></tr>
<tr><td>Fever
☐Present☐ Absent</td><td colspan="3">Wheezing
☐ Rhonchi (coarse crackles with secretions) ☐ Tachypnea (rapid shallow breathing) ☐ Stridor (monophonic inspiratory sound)
☐ Sibilant rhonchi (high-pitched whistling)</td></tr>
<tr><td colspan="4">Sputum Production
☐ Absent ☐ Present ☐ Yellow sputum ☐ White sticky sputum ☐ White frothy sputum ☐ Yellow-green purulent sputum</td></tr>
<tr><td colspan="2">Snoring
☐ Present ☐ Absent</td><td colspan="2">Rubbing Nose
☐ Present ☐ Absent</td></tr>
<tr><td>Nausea
☐Present☐Absent</td><td>Vomiting
☐Present☐Absent</td><td>Retrosternal Burning Sensation
☐Present☐Absent</td><td>Otalgia and Tinnitus
☐Present☐Absent</td></tr>
</table>

Physical Examination and Diagnostic Studies
**Skin rash** ☐Present ☐Absent
**Conjunctival congestion** ☐Present ☐Absent
**Infraorbital dark shadows** ☐Present ☐Absent
**Nose (nasal mucosa, saluting sign)**<br>☐Mucosal congestion and swelling ☐Thick/viscous secretions ☐Normal
**Condition of pharynx**<br>☐Erythema/congestion ☐Tonsillar hypertrophy<br>☐Follicular hyperplasia ☐Mucoid/purulent exudate coating ☐ Normal examination
**Pulmonary breath sounds**<br>☐Stridor ☐Wheezes ☐ Sonorous Rhonchi<br>☐ Gurgling Sounds ☐Crackles/Rales ☐No adventitious sounds
**Heart murmur** ☐Present ☐Absent
**Epigastric tenderness** ☐Present ☐Absent
**Abnormal bowel sounds** ☐ Abnormal ☐ Normal

Patient ID：|__|__|__|__| __|　　Initial：|__|__|__|__

Optional Test Results		
**Complete Blood Count (CBC)**<br>☐ Not documented☐ Within normal limits<br>☐ Abnormal results -Specify abnormalities:		
**CRP** ☐ Not documented ☐ Within normal limits ☐ Abnormal -Specify value:		
**ESR** ☐ Not documented ☐ Within normal limits ☐ Abnormal -Specify value:		
**PCT** ☐ Not documented ☐ Within normal limits ☐ Abnormal -Specify value:		
**SAA** ☐ Not documented ☐ Within normal limits ☐ Abnormal -Specify value:		
**T-SPOT** ☐ Not documented ☐ Within normal limits ☐ Abnormal -Specify value:		
**MP-Ab** ☐ Not documented ☐ Within normal limits ☐ Abnormal -Specify value:		
**IgE** ☐ Not documented ☐ Within normal limits ☐ Abnormal -Specify value:		
**Cardiac Enzymes**<br>☐ Not documented ☐ Within normal limits ☐ Abnormal -Specify value:		
**Troponin** ☐ Not documented ☐ Within normal limits ☐ Abnormal -Specify value:		
**Cellular Immunity** ☐ Not documented ☐ Within normal limits<br>☐ Abnormal -Specify value:		
**Humoral Immunity** ☐ Not documented ☐ Within normal limits<br>☐ Abnormal -Specify value:		
**Inhaled & Food Allergen Screening** ☐ Not documented ☐ Within normal limits<br>☐ Abnormal -Specify value:		
**Pulmonary Ventilation Function Parameters**<br>☐ Not documented ☐ Within normal limits ☐ Abnormal<br>FEV1 measured/predicted (%) FEV1/Vcmax(%) FVC measured/predicted (%)<br>FVC measured/predicted (%) VT(ml/kg) TPTEF/TE TPTEF/VE		
**Bronchodilation Test (BDT)**<br>☐ Not documented ☐ Within normal limits<br>☐ Abnormal -Improvement rate：		
FeNO☐ ☐<br>☐Not documented	CaNO☐ ☐<br>☐Not documented	FnNO☐ ☐<br>☐Not documented
**Lung Radiograph**<br>☐ Not documented ☐ Within normal limits<br>☐ Abnormal -Specify value:		
**CT**<br>☐ Not documented ☐ Within normal limits<br>☐ Abnormal -Specify value:		

**Bronchoscopy Report** ☐ Not documented ☐ Within normal limits ☐ Abnormal<br><br>Endoscopic findings:<br><br>BALF cellular analysis:<br><br>BALF inflammatory cytokines :<br><br>BALF culture & sensitivity :<br><br>BALF mNGS pathogen detection:
**Electrocardiogram (ECG/EKG)**<br>☐ Not documented ☐ Within normal limits<br>☐ Abnormal -Specify value:
**Echocardiography**<br>☐ Not documented ☐ Within normal limits<br>☐ Abnormal -Specify value:

Patient ID：|__|__|__|__|__| Initial：|__|__|__|__

Current Diagnosis
☐ Cough variant asthma (CVA) ☐ Uncontrolled asthma ☐ Asthma with co-existing infection ☐ Upper airway cough syndrome (UACS) ☐ Protracted bacterial bronchitis (PBB) ☐ Protracted pneumonia ☐ Allergic cough ☐Pulmonary fungal infection☐ Pulmonary fungal hypersensitivity ☐ Eosinophilic bronchitis (EB) ☐ Otic cough (Arnold's nerve reflex) ☐ Psychogenic cough ☐ Bronchial foreign body aspiration ☐ Congenital laryngotracheal/peritracheal vascular malformations ☐ Gastroesophageal reflux-induced cough ☐Drug-induced cough
**Treatment Plans (Name, Type, Dosage, Duration)**
**Follow-up (Closed-loop Management: Assessment → Diagnosis → Treatment → Reassessment)**
**Initial Follow-up Visit (After Weeks of Treatment)**<br>**Cough symptom at daytime**<br>☐No ☐ Brief cough episode ☐ Frequent coughing, slightly affecting daily life<br>☐ Frequent coughing, seriously affecting daily life<br>**Cough symptom at night**<br>☐ No ☐ Brief cough during sleep ☐ Coughing slightly affects sleep<br>☐ Coughing seriously affects sleep<br>**Auxiliary Examinations and Results:**<br><br>**Revised Diagnosis:**<br><br>**Continuation or Modification of Treatment Plan:**

**Subsequent Follow-up or Treatment Discontinuation (After Weeks of Treatment)**<br>**Cough symptom at daytime**<br>☐No ☐ Brief cough episode ☐ Frequent coughing, slightly affecting daily life<br>☐ Frequent coughing, seriously affecting daily life<br>**Cough symptom at night**<br>☐ No ☐ Brief cough during sleep ☐ Coughing slightly affects sleep<br>☐ Coughing seriously affects sleep<br>**Auxiliary Examinations and Results:**<br><br>**Revised Diagnosis:**<br><br>**Continuation or Modification of Treatment Plan:**
**Subsequent Follow-up Visit or Therapy termination (After Weeks of Treatment)**<br>**Cough symptom at daytime**<br>☐No ☐ Brief cough episode ☐ Frequent coughing, slightly affecting daily life<br>☐ Frequent coughing, seriously affecting daily life<br>**Cough symptom at night**<br>☐ No ☐ Brief cough during sleep ☐ Coughing slightly affects sleep<br>☐ Coughing seriously affects sleep

Patient ID：|__|__|__|__|__|　　　Initial：|__|__|__|__

Final diagnosis
☐ Cough variant asthma (CVA) ☐ Uncontrolled asthma ☐ Asthma with co-existing infection ☐ Upper airway cough syndrome (UACS) ☐ Protracted bacterial bronchitis (PBB) ☐ Protracted pneumonia ☐ Allergic cough ☐Pulmonary fungal infection☐ Pulmonary fungal hypersensitivity ☐ Eosinophilic bronchitis (EB) ☐ Otic cough (Arnold's nerve reflex) ☐ Psychogenic cough ☐ Bronchial foreign body aspiration ☐ Congenital laryngotracheal/peritracheal vascular malformations ☐ Gastroesophageal reflux-induced cough ☐Drug-induced cough
**Total Duration of Hospitalization at This Institution：**
**Causal Analysis of Protracted Disease Course：**

Registrant's Signature for the Study Above： ______________________

Signature Date：|__|__|__|__| Year |__|__| Month |__|__| Day
