# Supplementary material for: Analysis of Cough Factors and Quality of Life Score Among Children With Protracted Bacterial Bronchitis: Cross-Sectional Study
Source: JMIR Pediatr Parent. 2025 Dec 19;8:e82887. doi: 10.2196/82887 (PMC12716831; doi:10.2196/82887)
Supplement: Multimedia Appendix 2 [file pediatrics-v8-e82887-s002.pdf]

Table S1. 简化咳嗽症状积分表 (sCSS)

分值	日间咳嗽症状积分	夜间咳嗽症状积分
0	无咳嗽	无咳嗽
1	偶有短暂咳嗽	入睡时短暂咳嗽或偶有夜间咳嗽
2	频繁咳嗽，轻度影响日常活动	因咳嗽轻度影响夜间睡眠
3	频繁咳嗽，严重影响日常活动	因咳嗽严重影响夜间睡眠

Table S2. The cough symptom score (CSS)

Score	cough symptom at daytime	cough symptom at night
0	no cough during the day	no cough during the night
1	cough for one short period	cough on waking only
2	cough for more than two short periods	wake once or early due to cough
3	frequent coughing, which did not interfere with usual daytime activities	frequent waking due to coughs
4	frequent coughing, which did interfere with usual daytime activities	frequent coughs most of the night
5	distressing coughs most of the day	distressing coughs preventing any sleep

Table S3. Leicester Cough Questionnaire (LCQ)

This questionnaire is designed to assess the impact of cough on various aspects of your life. Read each question carefully and answer by CIRCLING the response that best applies to you. Please answer ALL questions, as honestly as you can.						
1. In the last 2 weeks, have you had chest or stomach pains as a result of your cough?						
1<br>All of the time	2<br>Most of the time	3<br>A good bit of the time	4<br>Some of the time	5<br>A little of the time	6<br>Hardly any of the time	7<br>None of the time
2. In the last 2 weeks, have you been bothered by sputum (phlegm) production when you cough?						
1<br>Every time	2<br>Most times	3<br>Several times	4<br>Some times	5<br>Occasionally	6<br>Rarely	7<br>Never
3. In the last 2 weeks, have you been tired because of your cough?						
1<br>All of the time	2<br>Most of the time	3<br>A good bit of the time	4<br>Some of the time	5<br>A little of the time	6<br>Hardly any of the time	7<br>None of the time
4. In the last 2 weeks, have you felt in control of your cough?						
1<br>None of the time	2<br>Hardly any of the time	3<br>A little of the time	4<br>Some of the time	5<br>A good bit of the time	6<br>Most of the time	7<br>All of the time
5. How often during the last 2 weeks have you felt embarrassed by your coughing?						
1<br>All of the time	2<br>Most of the time	3<br>A good bit of the time	4<br>Some of the time	5<br>A little of the time	6<br>Hardly any of the time	7<br>None of the time
6. In the last 2 weeks, my cough has made me feel anxious						
1<br>All of the time	2<br>Most of the time	3<br>A good bit of the time	4<br>Some of the time	5<br>A little of the time	6<br>Hardly any of the time	7<br>None of the time
7. In the last 2 weeks, my cough has interfered with my job, or other daily tasks						
1<br>All of the time	2<br>Most of the time	3<br>A good bit of the time	4<br>Some of the time	5<br>A little of the time	6<br>Hardly any of the time	7<br>None of the time

8. In the last 2 weeks, I felt that my cough interfered with the overall enjoyment of my life						
1<br>All of the time	2<br>Most of the time	3<br>A good bit of the time	4<br>Some of the time	5<br>A little of the time	6<br>Hardly any of the time	7<br>None of the time
9. In the last 2 weeks, exposure to paints or fumes has made me cough						
1<br>All of the time	2<br>Most of the time	3<br>A good bit of the time	4<br>Some of the time	5<br>A little of the time	6<br>Hardly any of the time	7<br>None of the time
10. In the last 2 weeks, has your cough disturbed your sleep?						
1<br>All of the time	2<br>Most of the time	3<br>A good bit of the time	4<br>Some of the time	5<br>A little of the time	6<br>Hardly any of the time	7<br>None of the time
11. In the last 2 weeks, how many times a day have you had coughing bouts?						
1<br>All of the time (continuously)	2<br>Most times during the day	3<br>Several times during the day	4<br>Some times during the day	5<br>Occasionally through the day	6<br>Rarely	7<br>None
12. In the last 2 weeks, my cough has made me feel frustrated						
1<br>All of the time	2<br>Most of the time	3<br>A good bit of the time	4<br>Some of the time	5<br>A little of the time	6<br>Hardly any of the time	7<br>None of the time
13. In the last 2 weeks, my cough has made me feel fed up						
1<br>All of the time	2<br>Most of the time	3<br>A good bit of the time	4<br>Some of the time	5<br>A little of the time	6<br>Hardly any of the time	7<br>None of the time
14. In the last 2 weeks, have you suffered from a hoarse voice as a result of your cough?						
1<br>All of the time	2<br>Most of the time	3<br>A good bit of the time	4<br>Some of the time	5<br>A little of the time	6<br>Hardly any of the time	7<br>None of the time

15. In the last 2 weeks, have you had a lot of energy?						
1<br>None of the time	2<br>Hardly any of the time	3<br>A little of the time	4<br>Some of the time	5<br>A good bit of the time	6<br>Most of the time	7<br>All of the time
16. In the last 2 weeks, have you worried that your cough may indicate serious illness?						
1<br>All of the time	2<br>Most of the time	3<br>A good bit of the time	4<br>Some of the time	5<br>A little of the time	6<br>Hardly any of the time	7<br>None of the time
17. In the last 2 weeks, have you been concerned that other people think something is wrong with you, because of your cough?						
1<br>All of the time	2<br>Most of the time	3<br>A good bit of the time	4<br>Some of the time	5<br>A little of the time	6<br>Hardly any of the time	7<br>None of the time
18. In the last 2 weeks, my cough has interrupted conversation or telephone calls						
1<br>Every time	2<br>Most of the time	3<br>A good bit of the time	4<br>Some of the time	5<br>A little of the time	6<br>Hardly any of the time	7<br>None of the time
19. In the last 2 weeks, I feel that my cough has annoyed my partner, family or friends						
1<br>Every time I cough	2<br>Most times when I cough	3<br>Several times when I cough	4<br>Some times when I cough	5<br>Occasionally when I cough	6<br>Rarely	7<br>Never
Thank you for completing this questionnaire.						
Note: A 7 point Likert scale was used in the LCQ ranging from 1=all of the time to 7=none of the time. A higher score indicated better health status. Domains were scored out of 7 (total score from items in domain/number of items in domain). The overall score for the LCQ for each patient was calculated by adding the individual domain scores.<br>Physical domain: items of 1, 2, 3, 9, 10, 11, 14, 15<br>Psychological domain: items of 4, 5, 6, 12, 13, 16, 17<br>Social domain: items of 7, 8, 18, 19						

Table S4.莱塞斯特咳嗽问卷普通话版（LCQ-MC）

这份问卷是为评估咳嗽对您生活各个方面的影响而设计的。请认真阅读每一个问题，并在最适合你情况的描述上画圈。请如实回答每个问题。

1. 在过去的两周内，您会因为咳嗽而胸痛或肚子痛吗?

①一直都会　②大多数时间会　③常常会　④有时会　⑤很少会　⑥几乎不会　⑦一点也不会

2. 在过去的两周内，您会因为咳嗽时有痰而感到烦恼吗?

①每次都会　②大多数时候会　③时时会　④有时会　⑤偶尔会　⑥极少会　⑦从来不会

3. 在过去的两周内，您会因为咳嗽而感到疲倦吗?

①一直都会　②大多数时间会　③常常会　④有时会　⑤很少会　⑥几乎不会　⑦一点也不会

4.在过去的两周内，您觉得能控制自己的咳嗽吗?

①一点也不能　②几乎不能 ③很少能　④有时能　⑤常常能　⑥大多数时间能　⑦一直都能

5.在过去的两周内，您会因为咳嗽而感到尴尬吗?

①一直都会　②大多数时间会　③常常会　④有时会　⑤很少会　⑥几乎不会　⑦一点也不会

6.在过去的两周内，您会因为咳嗽而焦虑不安吗?

①一直都会　②大多数时间会　③常常会　④有时会　⑤很少会　⑥几乎不会　⑦一点也不会

7.在过去的两周内，咳嗽会干扰您的工作或其他日常事务吗?

①一直都会　②大多数时间会　③常常会　④有时会　⑤很少会　⑥几乎不会　⑦一点也不会

8.在过去的两周内，咳嗽会干扰您的整个娱乐生活吗?

①一直都会　②大多数时间会　③常常会　④有时会　⑤很少会　⑥几乎不会　⑦一点也不会

9. 在过去的两周内，接触油漆或油烟会让您咳嗽吗?

①一直都会　②大多数时间会　③常常会　④有时会　⑤很少会　⑥几乎不会　⑦一点也不会

10.在过去的两周内，咳嗽会影响您的睡眠吗?

①一直都会　②大多数时间会　③常常会　④有时会　⑤很少会　⑥几乎不会　⑦一点也不会

11.在过去的两周内，您每天阵发性咳嗽的次数多吗?

①持续有 ②次数多 ③时时有 ④有一些 ⑤偶尔有 ⑥极少有 ⑦一点也没有

12.在过去的两周内，咳嗽会让您感到沮丧吗?

①一直都会 ②大多数时间会 ③常常会 ④有时会 ⑤很少会 ⑥几乎不会 ⑦一点也不会

13.在过去的两周内，咳嗽会让您感到厌烦吗?

①一直都会 ②大多数时间会 ③常常会 ④有时会 ⑤很少会 ⑥几乎不会 ⑦一点也不会

14.在过去的两周内，您会因为咳嗽而声音嘶哑吗?

① 一直都会 ②大多数时间会 ③常常会 ④有时会 ⑤很少会 ⑥几乎不会 ⑦一点也不会

15.在过去的两周内，您会觉得精力充沛吗?

① 一点也不会 ②几乎不会 ③很少会 ④有时会 ⑤常常会 ⑥多数时间会 ⑦一直都会

16.在过去的两周内，咳嗽会让您担心自己得了严重疾病吗?

①一直都会 ②大多数时间会 ③常常会 ④有时会 ⑤很少会 ⑥几乎不会 ⑦一点也不会

17.在过去的两周内，咳嗽会让您担心别人觉得您身体不对劲吗?

① 一直都会 ②大多数时间会 ③常常会 ④有时会 ⑤很少会 ⑥几乎不会 ⑦一点也不会

18.在过去的两周内，咳嗽会让您中断谈话或接听电话吗?

① 每次都会 ②大多数时候会 ③常常会 ④有时会 ⑤很少会 ⑥几乎不会 ⑦一点也不会

19.在过去的两周内，咳嗽会让您的同伴、家人或朋友不耐烦吗?

①每次都会 ②大多数时候会 ③时时会 ④有时会 ⑤偶尔会 ⑥极少会 ⑦从来不会

多谢您的合作！

LCQ 评分：

（1）维度（问题）

（a）生理：问题 1,2,3,9,10,11,14,15

（b）心理：问题 4,5,6,12,13,16,17

（c）社会：问题 7,8,18，19

（2）维度得分：维度内各个问题的分数之和/维度内问题数（得分范围：1-7）

（3）总分：三个维度分数相加（得分范围：3-21）

Table S5. Parent-Proxy Cough-specific quality-of-Life Questionnaire (PC-QOL)

This questionnaire is designed to assess the impact of a child's cough on the parent/caregiver as well as the parental perception of their child's QOL. Read each question carefully and answer by CIRCLING the response that best applies to you. Please answer **ALL** questions, as honestly as you can.						
**During the past week, how often**						
1. Did you feel frightened when your child experienced cough?						
1<br>All of the time	2<br>Most of the time	3<br>Quite often	4<br>Some of the time	5<br>Once in a while	6<br>Hardly any of the time	7<br>None of the time
2. Did you feel frustrated because of your child's cough?						
1<br>All of the time	2<br>Most of the time	3<br>Quite often	4<br>Some of the time	5<br>Once in a while	6<br>Hardly any of the time	7<br>None of the time
3. Did your child's cough interfere with your job or work around house?						
1<br>All of the time	2<br>Most of the time	3<br>Quite often	4<br>Some of the time	5<br>Once in a while	6<br>Hardly any of the time	7<br>None of the time
4. Did you feel upset because of your child's cough?						
1<br>All of the time	2<br>Most of the time	3<br>Quite often	4<br>Some of the time	5<br>Once in a while	6<br>Hardly any of the time	7<br>None of the time
5. Did you have sleepless nights because of your child's cough?						
1<br>All of the time	2<br>Most of the time	3<br>Quite often	4<br>Some of the time	5<br>Once in a while	6<br>Hardly any of the time	7<br>None of the time
6. Were you awakened during the night because of your child's cough?						
1<br>All of the time	2<br>Most of the time	3<br>Quite often	4<br>Some of the time	5<br>Once in a while	6<br>Hardly any of the time	7<br>None of the time
7. Did you feel anxious about your child's cough?						
1<br>All of the time	2<br>Most of the time	3<br>Quite often	4<br>Some of the time	5<br>Once in a while	6<br>Hardly any of the time	7<br>None of the time

8. Did you feel helpless because of your child's cough?						
1<br>All of the time	2<br>Most of the time	3<br>Quite often	4<br>Some of the time	5<br>Once in a while	6<br>Hardly any of the time	7<br>None of the time
9. Did you feel stressed because of your child's cough?						
1<br>All of the time	2<br>Most of the time	3<br>Quite often	4<br>Some of the time	5<br>Once in a while	6<br>Hardly any of the time	7<br>None of the time
10. Did you feel sad about your child's cough?						
1<br>All of the time	2<br>Most of the time	3<br>Quite often	4<br>Some of the time	5<br>Once in a while	6<br>Hardly any of the time	7<br>None of the time
11. Did you feel scared because of your child's cough?						
1<br>All of the time	2<br>Most of the time	3<br>Quite often	4<br>Some of the time	5<br>Once in a while	6<br>Hardly any of the time	7<br>None of the time
12. Did you feel tired or exhausted because of your child's cough?						
1<br>All of the time	2<br>Most of the time	3<br>Quite often	4<br>Some of the time	5<br>Once in a while	6<br>Hardly any of the time	7<br>None of the time
13. Did you feel being over protective because of your child's cough?						
1<br>All of the time	2<br>Most of the time	3<br>Quite often	4<br>Some of the time	5<br>Once in a while	6<br>Hardly any of the time	7<br>None of the time
14. Did you feel powerless because of your child's cough?						
1<br>All of the time	2<br>Most of the time	3<br>Quite often	4<br>Some of the time	5<br>Once in a while	6<br>Hardly any of the time	7<br>None of the time
15. Did you feel sorry for your child because of your child's cough?						
1<br>All of the time	2<br>Most of the time	3<br>Quite often	4<br>Some of the time	5<br>Once in a while	6<br>Hardly any of the time	7<br>None of the time

**During the past week, how worried or concerned were you?**						
16. About your child's performance of normal activities including feeding and schooling?						
1<br>Very, Very Worried/Concerned	2<br>Very Worried/Concerned	3<br>Fairly Worried/Concerned	4<br>Somewhat Worried/Concerned	5<br>A little Worried/Concerned	6<br>Hardly Worried/Concerned	7<br>Not Worried/Concerned
17. Over the medications your child takes and the side effects of them?						
1<br>Very, Very Worried/Concerned	2<br>Very Worried/Concerned	3<br>Fairly Worried/Concerned	4<br>Somewhat Worried/Concerned	5<br>A little Worried/Concerned	6<br>Hardly Worried/Concerned	7<br>Not Worried/Concerned
18. About your child being able to lead a normal life?						
1<br>Very, Very Worried/Concerned	2<br>Very Worried/Concerned	3<br>Fairly Worried/Concerned	4<br>Somewhat Worried/Concerned	5<br>A little Worried/Concerned	6<br>Hardly Worried/Concerned	7<br>Not Worried/Concerned
19. About the cause of your child's cough?						
1<br>Very, Very Worried/Concerned	2<br>Very Worried/Concerned	3<br>Fairly Worried/Concerned	4<br>Somewhat Worried/Concerned	5<br>A little Worried/Concerned	6<br>Hardly Worried/Concerned	7<br>Not Worried/Concerned
20. About your child's cough indicating a serious illness?						
1	2	3	4	5	6	7
Very, Very Worried/Concerned	Very Worried/Concerned	Fairly Worried/Concerned	Somewhat Worried/Concerned	A little Worried/Concerned	Hardly Worried/Concerned	Not Worried/Concerned
21. About your child choking when coughing?						
1	2	3	4	5	6	7
Very, Very Worried/Concerned	Very Worried/Concerned	Fairly Worried/Concerned	Somewhat Worried/Concerned	A little Worried/Concerned	Hardly Worried/Concerned	Not Worried/Concerned
22. About leaving your child with others because of his/her cough?						
1	2	3	4	5	6	7
Very, Very Worried/Concerned	Very Worried/Concerned	Fairly Worried/Concerned	Somewhat Worried/Concerned	A little Worried/Concerned	Hardly Worried/Concerned	Not Worried/Concerned
23. About your child not being able to breathe again after coughing?						
1<br>Very, Very Worried/Concerned	2<br>Very Worried/Concerned	3<br>Fairly Worried/Concerned	4<br>Somewhat Worried/Concerned	5<br>A little Worried/Concerned	6<br>Hardly Worried/Concerned	7<br>Not Worried/Concerned

24. About the effects of your child's cough on him/her?						
1<br>Very, Very Worried/Concerned	2<br>Very Worried/Concerned	3<br>Fairly Worried/Concerned	4<br>Somewhat Worried/Concerned	5<br>A little Worried/Concerned	6<br>Hardly Worried/Concerned	7<br>Not Worried/Concerned
25. About your child feeling tired because of the cough?						
1<br>Very, Very Worried/Concerned	2<br>Very Worried/Concerned	3<br>Fairly Worried/Concerned	4<br>Somewhat Worried/Concerned	5<br>A little Worried/Concerned	6<br>Hardly Worried/Concerned	7<br>Not Worried/Concerned
26. About your child not sleeping well because of the cough?						
1<br>Very, Very Worried/Concerned	2<br>Very Worried/Concerned	3<br>Fairly Worried/Concerned	4<br>Somewhat Worried/Concerned	5<br>A little Worried/Concerned	6<br>Hardly Worried/Concerned	7<br>Not Worried/Concerned
27. About the cough causing damage to your child's chest or lungs?						
1<br>Very, Very Worried/Concerned	2<br>Very Worried/Concerned	3<br>Fairly Worried/Concerned	4<br>Somewhat Worried/Concerned	5<br>A little Worried/Concerned	6<br>Hardly Worried/Concerned	7<br>Not Worried/Concerned
Thank you for completing this questionnaire.						
Note: A 7 point Likert scale was used in the PC-QOL ranging from 1=all of the time/ Very, Very Worried or Concerned to 7=none of the time/ Not Worried or Concerned. A higher score reflected greater frequency and greater concerns or worries. Domains were scored out of 7 (total score from items in domain/number of items in domain). The overall score for the PC-QOL for each patient was calculated by adding the individual domain scores.<br>Physical domain: items of 5, 6, 12, 17, 19, 20, 21, 23, 24, 26, 27<br>Psychological domain: items of 1, 2, 4, 7, 8, 9, 10, 11, 14, 15, 25<br>Social domain: items of 3, 13, 16, 18, 22						
