# Supplementary material for: Analysis of Cough Factors and Quality of Life Score Among Children With Protracted Bacterial Bronchitis: Cross-Sectional Study
Source: JMIR Pediatr Parent. 2025 Dec 19;8:e82887. doi: 10.2196/82887 (PMC12716831; doi:10.2196/82887)
Supplement: Multimedia Appendix 3 [file pediatrics-v8-e82887-s003.pdf]

Table S1. Characteristics of 88 children

	**N=88**
**Gender, Male, n (%)**	57(65)
**Age of onset (months), n (%)**	
0~12	5(6)
13~36	8(9)
37~72	48(55)
73~108	21(24)
>108	6(7)
**Duration of cough (weeks), n (%)**	
4~8	47(53)
9~12	18(21)
13~24	11(13)
25~48	8(9)
>48	4(5)
**Rural residence**	33(38)
**Siblings**	45(51)
**Parents with high school education level or below**	25(28)
**Environmental exposure to smoking**	14(16)
**Did not rest at home after disease development**	55(63)
**Primary caregivers were grandparents**	21(24)
**Parents misjudged the nature of cough**	14(16)
**Number of visits to other hospitals, n (%)**	
0	2(2)
1~4	75(85)

5~9	9(10)
≥10	2(2)
**Medical institutions, n (%)**	
Hospitals at county level and below	45(51)
Municipal level and above hospitals	43(49)
**Diagnosis and treatment in other hospitals, n (%)**	
Cough variant asthma (CVA)	29(33)
Bronchitis/bronchopneumonia	57(65)
Antibiotic therapy	41(47)
Treatment with traditional Chinese medicine	20(23)
Family self-treatment	5(6)

Table S2. Usage of antibiotics outside the hospital

	N=73			
**Previous treatment with**	**Courses of treatment, n (%)**			
**Antibiotics**	≤1 week	1~2 weeks	≥ 2 weeks	Total
**Penicillins**	11(15)	5(7)	2(3)	18(25)
**Second generation Cephalosporins**	8(11)	2(3)	1(1)	11(15)
**Third generation Cephalosporins**	14(19)	4(5)	1(1)	19(26)
**Macrolides**	20(27)	8(11)	5(7)	33(45)

Table S3. Pathogen detection in the bronchoalveolar lavage fluid

	**N=39 (39/63,62%)**
**Positive cases, n (%)**	
Only bacteria	19(49%)
Bacteria + virus	18(36%)
Bacteria + fungi	2(5%)
**Number of bacteria, n (%)**	
1	26(67%)
2	10(26%)
3	3(8%)
**Bacterial species, n (%)**	
Streptococcus pneumoniae	17(44%)
Haemophilus influenzae	7(18%)
Moraxella catarrhalis	2(5%)
Staphylococcus aureus	6(15%)
Klebsiella pneumoniae	3(8%)
Pseudomonas aeruginosa	1(3%)

Table S4. Antibiotic use (N=88)

Antibiotic Type	Cases	Duration of Antibiotic Therapy(weeks),mean(SD)
Cefditoren Pivoxil Granules	47	2.7(0.75)
Faropenem Sodium Tablets	27	3.0 (1.11)
Linezolid Tablets	22	2.5 (1.33)

Table S5. Duration of antimicrobial therapy (N=88)

Duration	Cases, n (%)
2 weeks	27(31)
3 weeks	29(33)
4 weeks	24(27)
5 weeks	2(2)
6 weeks	5(6)
8 weeks	1(1)

Table S6. sCSS[a] at first visit(N=88)

sCSS	Cases, n (%)
**Cough during the day**	
0	4(5)
1	66(75)
2	15(17)
3	3(3)
**Cough at night**	
0	20(23)
1	40(45)
2	24(27)
3	4(5)

[a] **sCSS**: Simplified Cough Symptom Score

Table S7. Comparison of cough symptom scores between day and night(N=88)

Phase of cough	Scores, mean(IQR)	*z*	*P* value
**Day**	1(IQR 1-1)	-0.52	.60
**Night**	1(IQR 1-2)		

Table S8. Efficacy evaluation of daytime cough(N=88)

Curative effect	Followed for 2 weeks<br>Cases, n (%)	Followed for 4 weeks<br>Cases, n (%)
**Cure**	33(38)	79(90)
**Improve**	12(14)	2(2)
**No response**	37(42)	6(7)
**Aggravating**	6(7)	1(1)

Table S9. Efficacy evaluation of nocturnal cough(N=88)

Curative effect	Followed for 4 weeks Cases, n (%)	Followed for 4 weeks Cases, n (%)
**Cure**	62(70)	86(98)
**Improve**	12(14)	1(1)
**No response**	12(14)	1(1)
**Aggravating**	2(2)	0(0)

Table S10. LCQ-MC[a] score (N=39)

Domain	Scores, mean (SD)
**Physical**	4.90 (0.89)
**Psychological**	4.99 (1.28)
**Social**	4.85 (1.37)
**Sum**	14.74 (3.11)

[a] **LCQ-MC:** Leicester Cough Questionnaire in Mandarin-Chinese

Table S11. The reliability and validity of PC-QOL[a] scale (N=88)

Domain	Cronbach α	KMO	Scores, mean (SD)
**Physical**	0.92	0.89	3.10 (1.36)
**Psychological**	0.95	0.91	3.32 (1.57)
**Social**	0.81	0.81	3.67 (1.53)
**Sum**	0.97	0.90	10.09 (4.21)

[a] **PC-QOL:** Parent-Proxy Cough-Specific Quality of Life

Table S12. Correlation analysis of LCQ-MC[a] and PC-QOL[b] scores among children aged ⩾6 years(N=35)

Domain	LCQ-MC, mean (SD)	PC-QOL, mean (SD)	*r*	*P* value
**Physical**	4.90 (0.89)	3.24 (1.43)	0.57	<.001
**Psychological**	4.99 (1.28)	3.38 (1.53)	0.48	.004
**Social**	4.85 (1.37)	3.73 (1.49)	0.58	<.001
**Sum**	14.74 (3.11)	10.35 (4.21)	0.66	<.001

[a] **LCQ-MC:** Leicester Cough Questionnaire in Mandarin-Chinese

[b] **PC-QOL:** Parent-Proxy Cough-Specific Quality of Life
